# Bioinformatics-based analysis of the relationship between STC1 expression and immune infiltration in gastric cancer

**DOI:** 10.3389/fgene.2025.1499121

**Published:** 2025-07-16

**Authors:** Weijun Ma, Xiaoli Ma, Yaoqi Li

**Affiliations:** ^1^ Department of Ultrasound, The First Hospital of Lanzhou University, Lanzhou, China; ^2^ The Department of Rehabilitation, Gansu Provincial Maternity and Child-Care Hospital (Gansu Provincial Center Hospital), Lanzhou, China; ^3^ Department of Surgical Oncology, Gansu Provincial Hospital, Lanzhou, China

**Keywords:** gastric cancer, Stanniocalcin 1, prognosis, TME, T-cell exhaustion

## Abstract

**Objective:**

The study assessed the expression of STC1, and its potential as an immunotherapy target in gastric cancer (GC).

**Methods:**

RNAseq data from the TCGA_STAD project and TCGA-GTEx project of UCSC XENA website and microarray data from GEO (GSE66229) were downloaded. Differential STC1 mRNA expression between GC and non-carcinoma tissues was examined. Its relation to clinicopathological characteristics and prognosis was evaluated using univariate and multivariate Cox regression. Tumor infiltrating immune cells (TIICs) in the tumor microenvironment (TME) were assessed by ssGSEA, and T-cell exhaustion by TIMER. Single-cell RNA sequencing (scRNA-seq) analysis was performed to validate the correlation between STC1 expression and T-cell exhaustion markers. Gene Set Enrichment Analysis (GSEA) was also conducted to explore potential functions of STC1 in GC.

**Results:**

STC1 expression was significantly higher in GC tissues and independently predicted poor overall survival. STC1 expression was related to patient T-stage, location, and overall survival events. Immune infiltration analysis showed significant associations between STC1 and multiple immune cells in TME, with strong links to T-cell exhaustion. scRNA-seq analysis confirmed the co-expression of STC1 with T-cell exhaustion markers in specific cell clusters. GSEA identified several signaling pathways, such as “SIGNALING_BY_INTERLEUKINS” and “JAK_STAT_SIGNALING_PATHWAY,” linked to STC1 expression in GC.

**Conclusion:**

STC1 is a promising target for immunotherapy in GC, as its expression is correlated with patient prognosis, immune infiltration, and T-cell exhaustion.

## 1 Introduction

Gastric cancer (GC) is the fifth most prevalent cancer globally, with high morbidity and mortality rates among malignant tumors (Sung et al., 2021). The development of GC is linked to genetics, *Helicobacter pylori* infection and environmental factors (Smyth et al., 2020). Despite surgical resection, the 5-year survival rate is about 42.9%, and long-term survival remains low (Li et al., 2022; Agnes et al., 2020).

Immunotherapy, including cancer vaccines, immune checkpoint inhibitors (ICI), and chimeric antigen receptor T cells, has shown great success in clinical trials (Robert, 2020; Ott et al., 2019). Studies indicate that combining trastuzumab with chemotherapy improves the survival rate of patients afflicted by HER2-positive GC (Meric-Bernstam et al., 2019), while pablizumab has been approved by the FDA for treating PD-L1 positive GC (Kim et al., 2018). However, only a fraction of patients benefit from these treatments, highlighting the need for effective therapeutic targets and prognostic markers.

Stanniocalcin 1 (STC1) is a glycoprotein that regulates calcium and phosphorus levels and is expressed in various tissues (Sundell et al., 1992). Recently, it has been discovered that STC1 is expressed in high levels in ovarian cancer cell lines and ovarian cancer tissues, leading to increased anti-apoptotic proteins and cell cycle regulatory proteins in normal or malignant ovarian cells that have overexpressed STC1 (Liu et al., 2010). In lung cancer, STC1 expression is negatively correlated with immunotherapeutic effects and contributes to anti-tumor immune cell activity in the tumor microenvironment (TME) by affecting tumor immune evasion and immunotherapy resistance (Lin et al., 2021). Hou et al. (2021) found STC1 expression to be high among breast cancer cases, to be related to poorer survival, to promote cancer cell growth and invasion *in vitro*, decrease radiation-induced apoptosis, and reduce cancer cell sensitivity to cisplatin. Additionally, it has been demonstrated that co-culture with platelet-derived growth factor-stimulated tumor-associated fibroblasts (CAFs) promotes migration and invasion of colon cancer cells mediated by STC1 (Peña et al., 2013). However, the role of STC1 in GC remains unclear. This study aims to explore the potential of STC1 as a therapeutic target and prognostic marker in GC by examining public databases.

## 2 Materials and methods

### 2.1 Data source

RNA-sequencing data of HTSeq-FPKM format for STAD project, along with clinical information, were obtained in TCGA (https://portal.gdc.cancer.gov/) database. The dataset comprised of 375 tumor tissues and 32 paraneoplastic tissues. In addition, RNA-sequencing data of FPKM format were transformed to transcripts per million reads (TPM) format and transformed to logarithmic scale. RNA sequencing data for TCGA and GTEx, preprocessed uniformly using the Toil process (Vivian et al., 2017), were acquired in UCSC XENA (https://xenabrowser.net/datapages/) website in TPM format. Additionally, GC and paraneoplastic tissue microarray expression data for the GSE66229 dataset were obtained from the GEO database.

### 2.2 Relationship between STC1 expression and clinicopathological features of GC

We employed the Wilcoxon rank sum test for comparing STC1 mRNA expression levels between various clinicopathological features, like age, gender, pathological grade, TNM stage, tumor site, residual tumor (R) classification, type of samples, and survival status. The obtained results were visualized using the “ggplot2” package (Ginestet, 2011).

### 2.3 Survival analysis of GC patients

By collecting clinical and pathological information from patients with GC and excluding incomplete sample data, we combined the information with STC1 expression data. We employed a data-driven approach to determine the optimal cutoff for STC1 expression using the “survminer” package. This approach ensures that the cutoff maximizes the distinction between survival outcomes for patients in high and low STC1 expression groups. Kaplan-Meier survival curves were generated for each group, and the differences between groups were compared using the Log-rank test. To investigate whether STC1 expression and additional clinical factors was significant for patient prognosis, both univariate and multivariate Cox regression analyses were executed. To account for multiple comparisons, Bonferroni correction was applied to adjust the p-values in the multivariate analysis to reduce the risk of false positives. The results were depicted using forest plots. A nomogram was used furthermore for predicting survival probability among GC patients.

### 2.4 Correlation between STC1 expression and TME immune infiltration as well as T-cell exhaustion

The infiltration levels of 24 TIILs (Bindea et al., 2013) were assessed using the “GSVA” (Hänzelmann et al., 2013) package and the “ssGSEA” (Barbie et al., 2009) algorithm, and the relation of STC1 expression levels with TIILs infiltration degrees was compared using Spearman analysis and further analyzed by TIMER (Li et al., 2017) database (https://cistrome.shinyapps.io/timer/) for further analyzing the relation of STC1 expression with T-cell exhaustion markers.

### 2.5 Single-cell RNA sequencing analysis

Single-cell RNA sequencing data were processed using Seurat (version 4.0). The percentage of mitochondrial genes was calculated using the PercentageFeatureSet function. Quality control steps included filtering out cells with fewer than 50 features or greater than 5% mitochondrial gene content. Data normalization was performed using the LogNormalize method. Highly variable genes were identified with the “vst” method. Principal component analysis (PCA) was conducted on the top 1,500 variable genes, and the top 20 principal components (PCs) were selected for subsequent clustering and dimensionality reduction. Clustering was performed using the FindNeighbors and FindClusters functions based on the first 10 PCs, with the resolution parameter set to 0.5. Clustering results were visualized using t-distributed stochastic neighbor embedding (t-SNE), and marker genes for each cluster were identified using the FindAllMarkers function. Cell types were annotated using the SingleR package, referencing the Human Primary Cell Atlas. Differential expression analysis was performed to identify significant markers, with criteria set at |log2 fold change| > 1 and adjusted p-value <0.05.

### 2.6 Gene enrichment analysis

We utilized the “DESeq2” (Love et al., 2014) package to identify differentially expressed genes based on STC1 expression grouping, followed by screening and display of differentially expressed genes with |log2(FC)| > 1 and p-adj <0.05 using volcano plots. We further utilized the “clusterProfiler” (Yu et al., 2012) and “ggplot2” (Ginestet, 2011) packages to perform GSEA analysis on the differentially expressed genes. For GSEA, gene sets were considered significantly enriched when the FDR-adjusted p-value was <0.05. All analyses were conducted using R software version 4.2.1.

## 3 Results

### 3.1 Baseline information of GC patients

Altogether 375 cases possessing the necessary clinical characteristics were obtained from the TCGA database. Statistics were categorized, based on age, gender, TNM stage, tissue grade, site of tumorigenesis, residual tumor classification, and patient survival status, and any missing information was excluded from (Table 1).

**TABLE 1:** Baseline information for patients with gastric cancer in TCGA database.

Characteristic	Levels	Overall
n		375
Age, n (%)	<=65	164 (44.2%)
	>65	207 (55.8%)
Gender, n (%)	Female	134 (35.7%)
	Male	241 (64.3%)
Pathologic stage, n (%)	Stage I&II	164 (46.6%)
	Stage III&IV	188 (53.4%)
T stage, n (%)	T1&2	99 (27.0%)
	T3&4	268 (73.0%)
N stage, n (%)	N0	111 (31.1%)
	N1&2&3	246 (68.9%)
M stage, n (%)	M0	330 (93%)
	M1	25 (7%)
Histologic grade, n (%)	G1&2	147 (40.1%)
	G3	219 (59.9%)
Location, n (%)	Distal	138 (33.9%)
	Non-distal	223 (66.1%)
Residual tumor, n (%)	R0	298 (90.6%)
	R1&2	31 (9.4%)
OS event, n (%)	Alive	228 (60.8%)
	Dead	147 (39.2%)

### 3.2 Differences in STC1 expression between GC and non-carcinoma tissues

**FIGURE 1:**
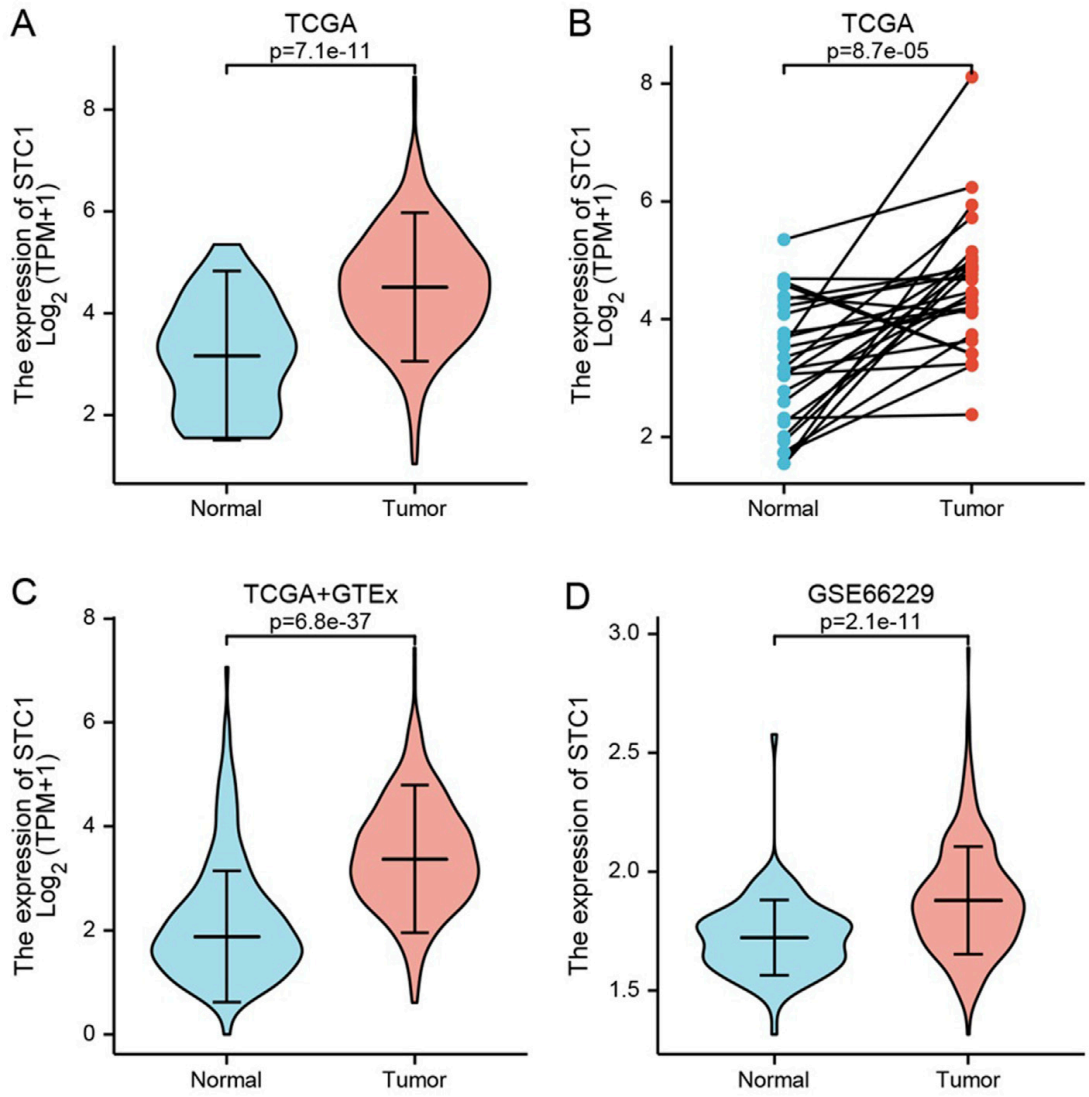
STC1 expression between gastric cancer and non-carcinoma tissues. **(A,B)** Differential STC1 expression between tumor and non-carcinoma tissues in TCGA database. **(C)** Differential STC1 expression between tumor and normal tissues in the combined TCGA and GTEx databases. **(D)** Differential STC1 expression between tumor and non-carcinoma tissues in GEO database.

Differentially expressed genes between GC tissues and paraneoplastic tissues in the TCGA database were summarized in Supplementary Table 1. Among these, STC1 expression was significantly elevated in GC tissues than in paraneoplastic tissues (P = 7.1e-11) (Figure 1A). To account for individual differences, we performed paired difference analysis between GC and corresponding paraneoplastic samples, which revealed that STC1 was highly expressed in GC tissues (P = 8.7e-05) (Figure 1B). Since there were only a few paracancer samples in the TCGA database, we downloaded RNAseq data of TPM format at UCSC XENA website based on Toil process for both TCGA and GTEx. We extracted STAD from TCGA and non-carcinoma sample data from GTEx, analyzed STC1 expression difference between the two groups, and discovered the high expression of STC1 within GC tissues (P = 6.8e-37) (Figure 1C). Furthermore, the GEO-derived results confirmed the high expression of STC1 within GC (P = 2.1e-11) (Figure 1D).

### 3.3 Relationship between STC1 expression and clinical factors

**FIGURE 2:**
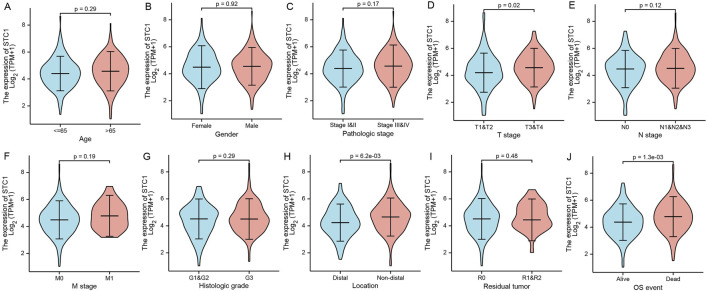
Relation of STC1 expression with clinical features in gastric cancer patients. **(A–J)** Differential expression of STC1 at different levels of age **(A)**, gender **(B)**, pathological stage **(C)**, T stage **(D)**, N stage **(E)**, M stage **(F)**, tissue grade **(G)**, tumor location **(H)**, residual tumor classification **(I)**, and survival status **(J)**, respectively.

Differential analysis of STC1 expression across clinical characteristics indicated that STC1 was expressed at higher levels in T3-4 stage, non-distal GC and death samples than in T1-2 stage (P = 0.02), Distal GC (P = 0.02), and in surviving patients (P = 1.3e-03), respectively. However, STC1 expression was not significantly different based on age, sex, M and N stage, and tissue grading. Similarly, residual tumor classification did not indicate any significant differences (Figures 2A-J).

### 3.4 Significance of STC1 in the prognosis prediction for GC patients

**FIGURE 3:**
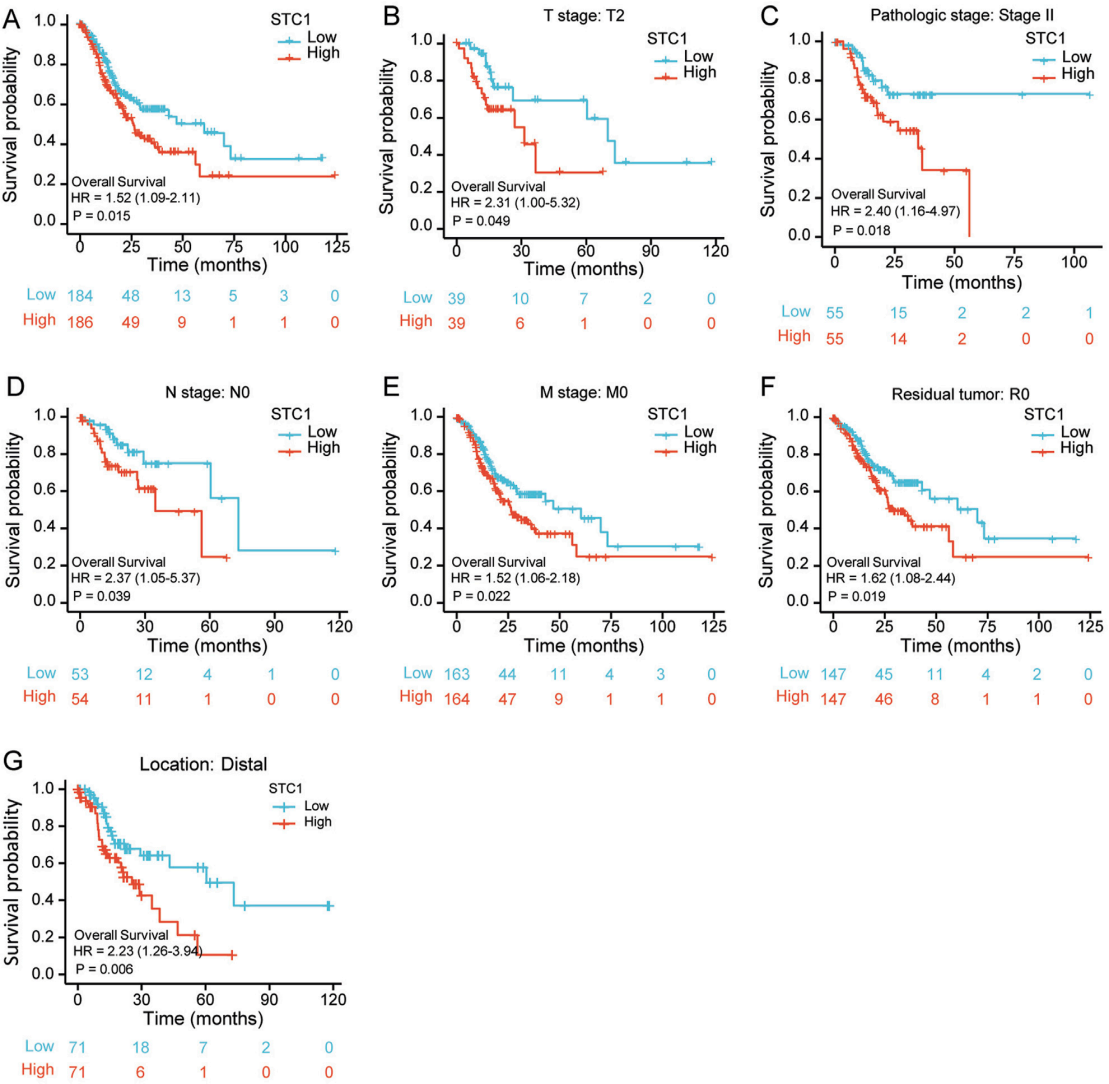
Relation of STC1 expression with prognosis in gastric cancer patients. **(A)** Differences in overall survival between high and low STC1 expression groups. **(B–G)** Differences in overall survival between high and low STC1 expression groups at different factor levels of T2, Stage II, N0, M0, R0, and Location.

**FIGURE 4:**
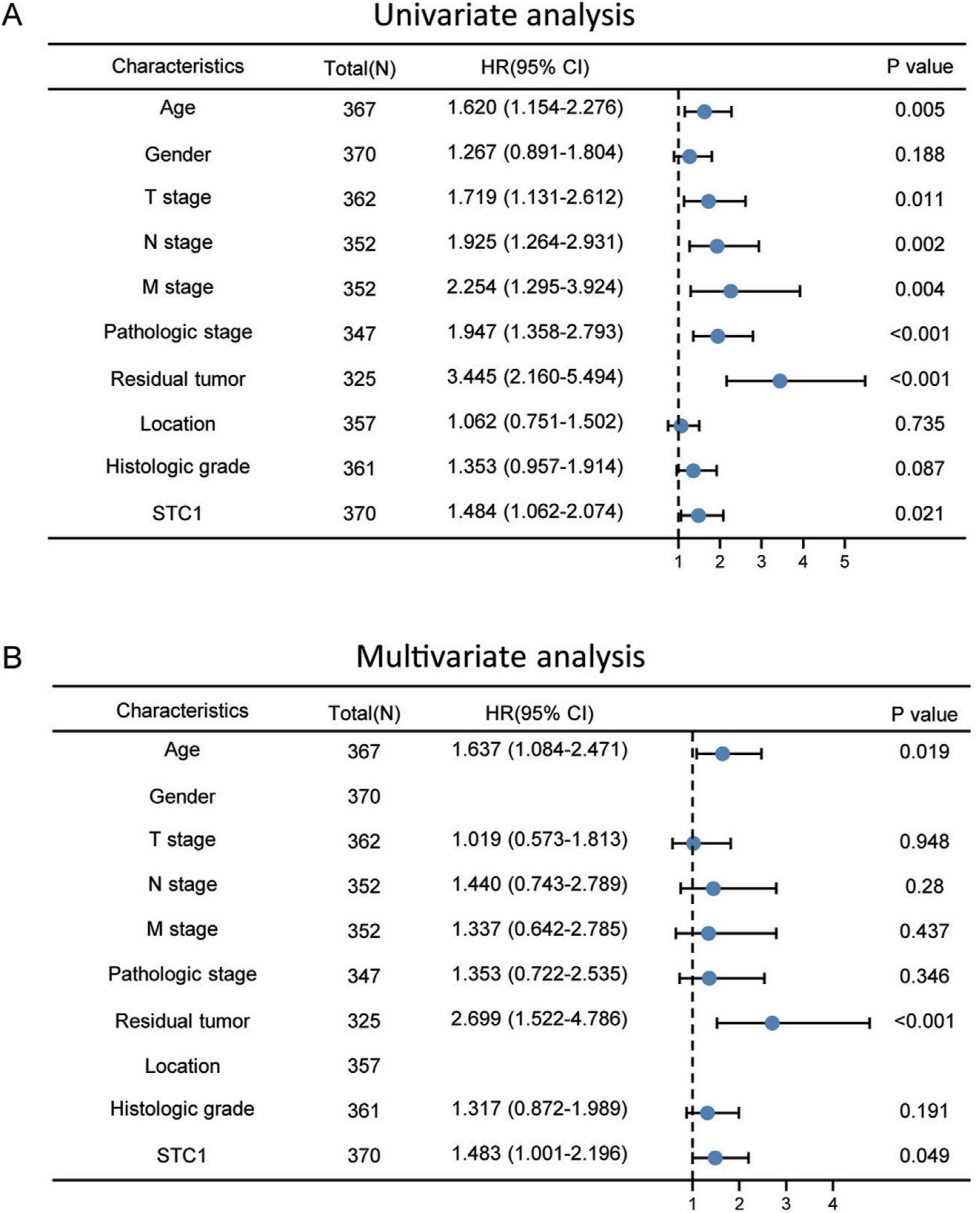
Univariate and multivariate Cox regression analyses. **(A)** Univariate **(B)** multivariate analysis.

**FIGURE 5:**
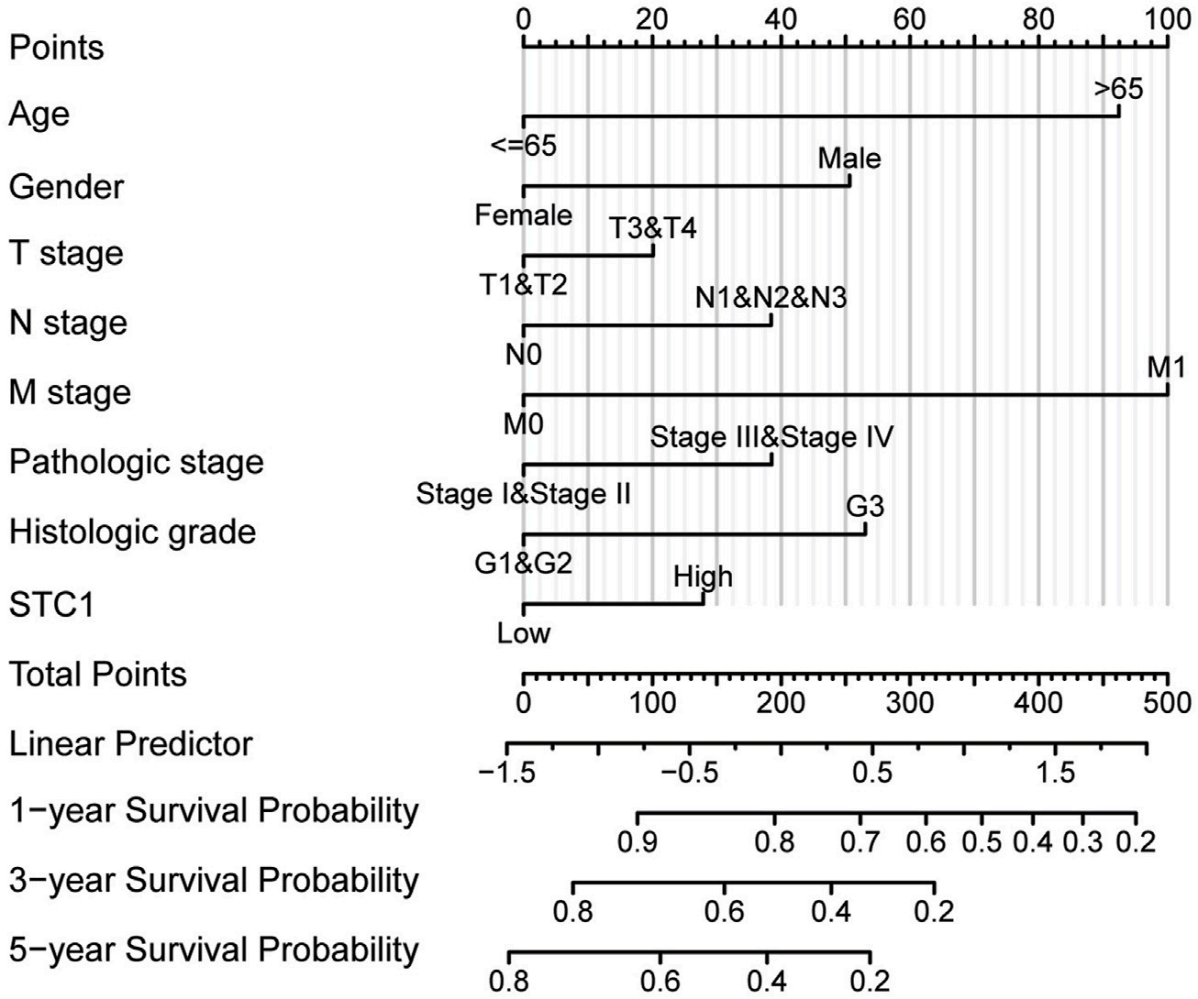
Nomogram for the prediction of 1-, 3- and 5-year survival probabilities.

According to Kaplan-Meier survival analysis, high STC1 expression group had significantly lower overall survival (OS) (P = 0.015) than low expression cohort of STC1 (Figure 3A). Subgroup analyses based on different clinical features demonstrated that elevated STC1 expression exhibited a significant relation to the dismal prognostic outcome in T2, stage II, N0, M0, R0, and Distal groups (Figures 3B–G). As revealed by univariate Cox regression, patient age, TNM stage, and STC1 expression all exhibited significant correlation with poor prognosis, whereas upon multivariate Cox regression, age, residual tumor characteristics, and STC1 expression were independent risk factors for OS in GC patients (Figures 4A,B). Additionally, a nomogram was constructed by incorporating age, gender, TNM stage, tissue grading, and STC1 expression level variables, and weight coefficients were separately calculated through Cox regression analysis. Based on the results, the summed scores of each variable were used for predicting 1, 3, and 5-year survival probabilities of GC cases. The smaller the total points, the higher the probability of survival (Figure 5).

### 3.5 Association between STC1 expression and TME immune infiltration as well as T-cell exhaustion

**FIGURE 6:**
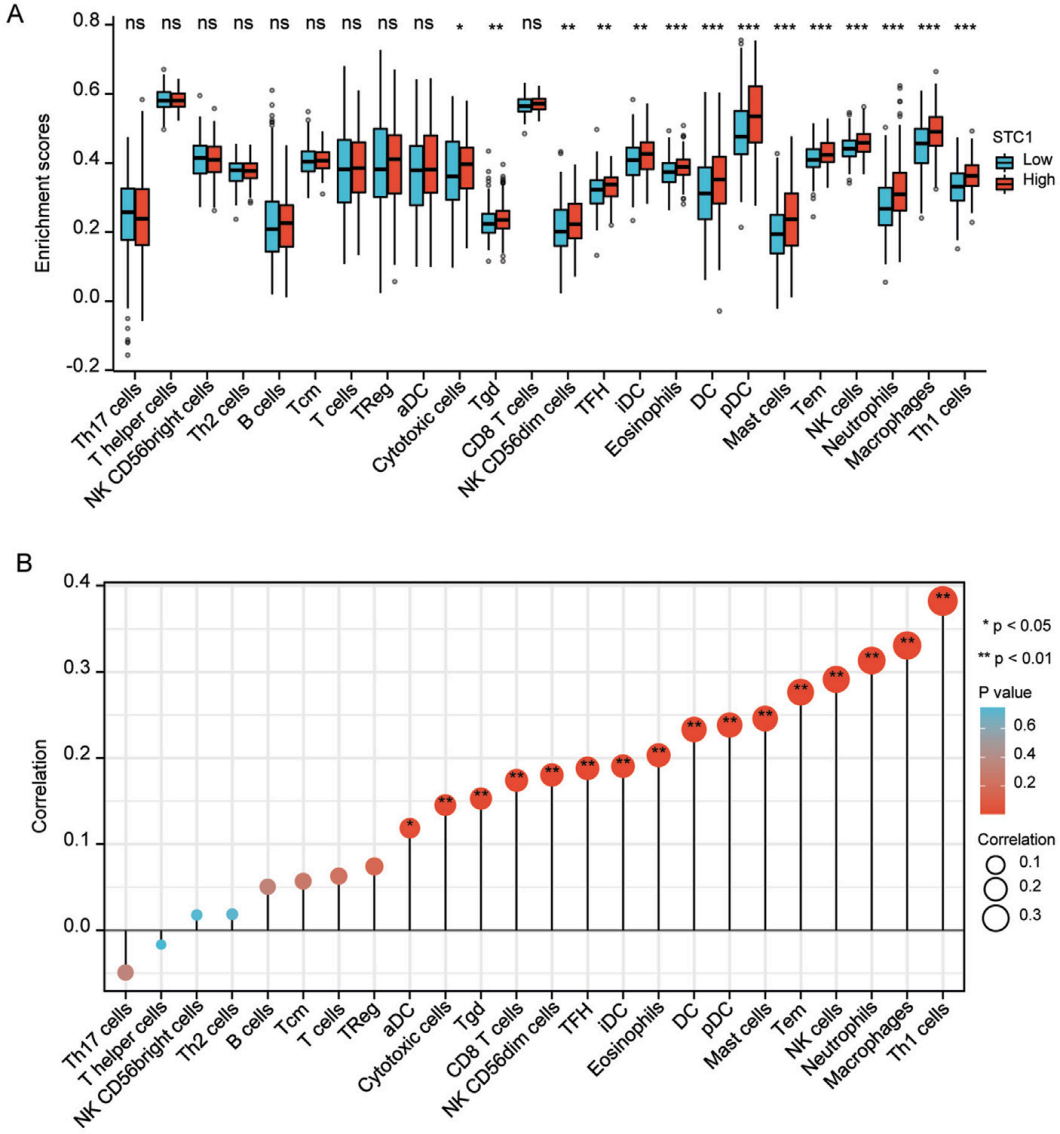
Relationship of STC1 expression with immune cell infiltration within gastric cancer tissues. **(A)** Differential STC1 expression within different TIICs. **(B)** Correlation of STC1 expression with TIIC.

**FIGURE 7:**
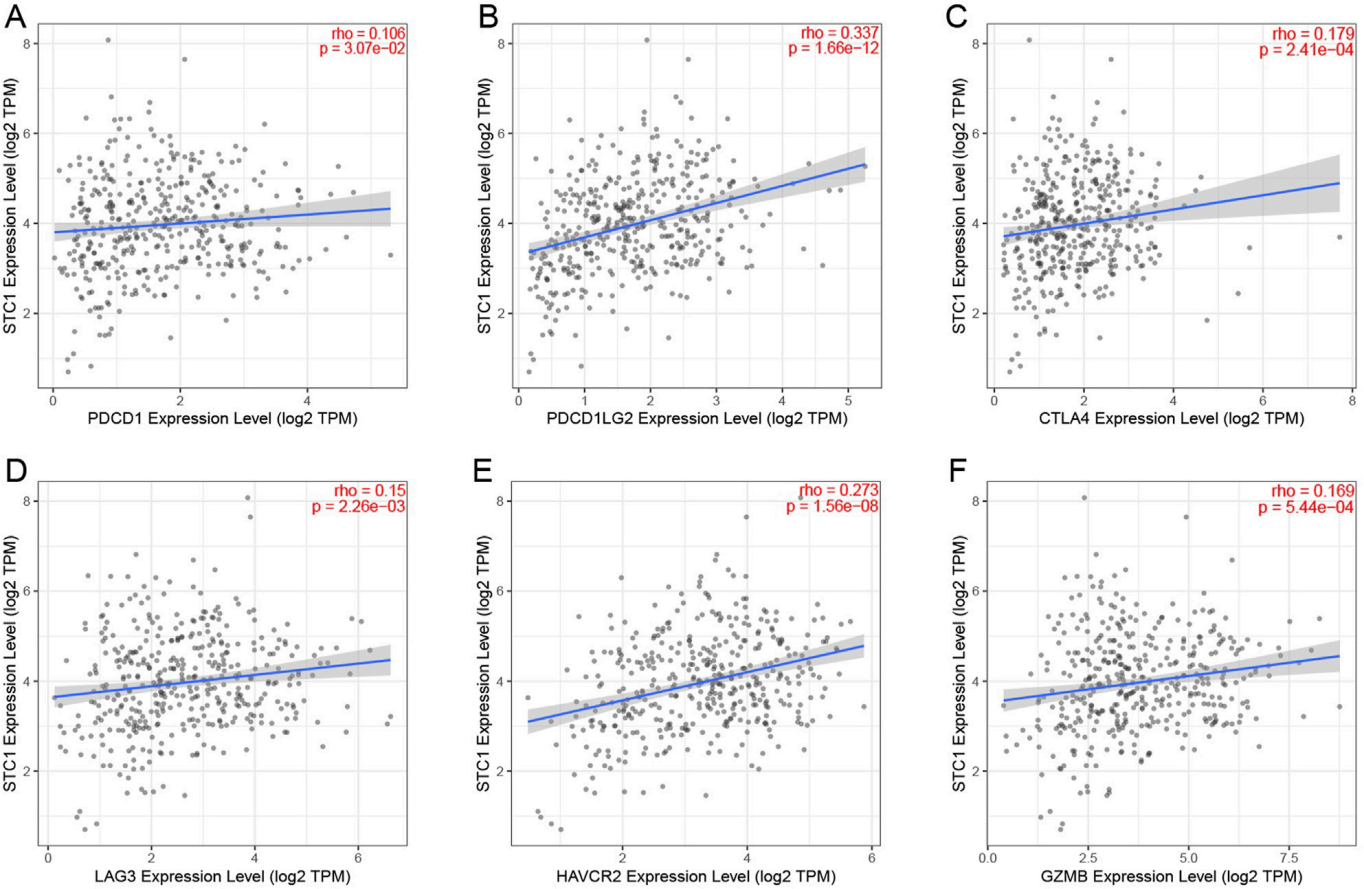
Relationship between STC1 expression and immunotherapy-related biomarkers. **(A–F)** Correlation of STC1 expression with the expression levels of T-cell exhaustion markers PDCD1 (PD-1), PDCD1LG2 (PD-L1), CTLA-4, LAG3, HAVCR2 (TIM3), and GZMB, respectively.

For the high expression cohort of STC1, there was a significant increase in the enrichment degrees of type 1 helper T cells, macrophages, neutrophils, natural killer cells, effector memory T cells, mast cells, plasma-like dendritic cells, eosinophils, dendritic cells, follicular helper T cells, immature dendritic cells, CD56dim natural killer cells, cytotoxic cells, and γδ T cells (Table 2; Figure 6A). However, enrichment degrees of activated dendritic cells,CD8+ T cells, regulatory T cells, T cells, central memory T cells, type 2 helper T cells, B cells, helper T cells, CD56bright natural killer cells, and type 17 helper T cells were not significantly different between the two cohorts. Spearman correlation analysis further revealed that, except for central memory T cells, regulatory T cells, B cells, Th2 cells, CD56bright natural killer cells, Th cells, and Th17 cells, STC1 expression exhibited significant positive relation to enrichment degrees of remaining tumor-infiltrating immune cells (Figure 6B). Additionally, STC1 expression showed significant positive relation to T-cell exhaustion markers PDCD1 (PD-1), PDCD1LG2 (PD-L1), CTLA-4, LAG3, HAVCR2 (TIM3), and GZMB expression (Figures 7A-F). Overall, based on the above results, STC1 is involved in regulating T cell activity.

**TABLE 2:** Results of KEGG pathway enrichment analysis.

ID	Description	GeneRatio	BgRatio	pvalue	p.adjust	qvalue
hsa04657	IL-17 signaling pathway	9/47	94/8076	2.52e-09	3.55e-07	2.97e-07
hsa04060	Cytokine-cytokine receptor interaction	10/47	295/8076	5.66e-06	3.99e-04	3.34e-04
hsa05144	Malaria	5/47	50/8076	9.35e-06	4.39e-04	3.67e-04
hsa04630	JAK-STAT signaling pathway	7/47	162/8076	3.68e-05	0.001	0.001
hsa05323	Rheumatoid arthritis	5/47	93/8076	1.90e-04	0.005	0.004

**FIGURE 8:**
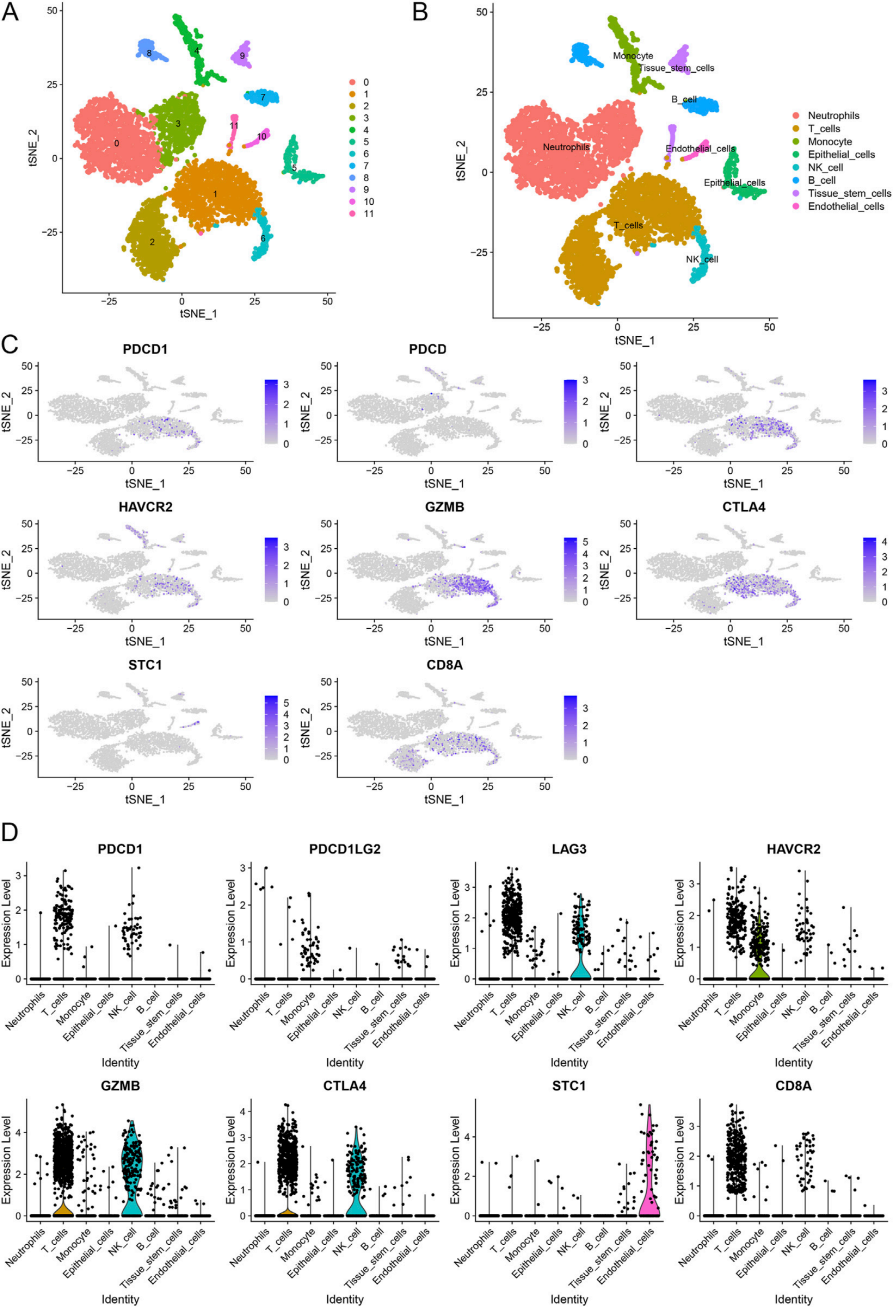
Single-cell RNA sequencing analysis of gastric cancer samples. **(A)** t-SNE plot illustrating the clustering of different cell types. **(B)** Annotation of cell clusters based on canonical markers. **(C)** t-SNE plots showing the expression of T-cell exhaustion markers and STC1 across different cell types. **(D)** Violin plots depicting the expression levels of T-cell exhaustion markers and STC1 in various cell types.

We performed scRNA-seq analysis to further investigate the correlation between STC1 expression and T-cell exhaustion markers in GC. The analysis revealed distinct clustering of various cell types, including neutrophils, T cells, monocytes, epithelial cells, NK cells, B cells, tissue stem cells, and endothelial cells (Figures 8A,B). We specifically focused on the expression patterns of PDCD1 (PD-1), PDCD1LG2 (PD-L1), HAVCR2 (TIM-3), GZMB, CTLA4 within these clusters. The t-SNE plots (Figure 8C) illustrate that PDCD1, PDCD1LG2, HAVCR2, and other exhaustion markers are predominantly expressed in T cells and epithelial cells. Notably, STC1 expression was observed in the same cell clusters, particularly those expressing high levels of T-cell exhaustion markers. This co-expression pattern indicates a potential relationship between elevated STC1 levels and an immunosuppressive tumor microenvironment in GC. The expression levels of these markers across different cell types are further quantified in violin plots (Figure 8D). These results confirm that STC1 overexpression is associated with cells exhibiting markers of T-cell exhaustion.

### 3.6 Pathway enrichment analysis

**FIGURE 9:**
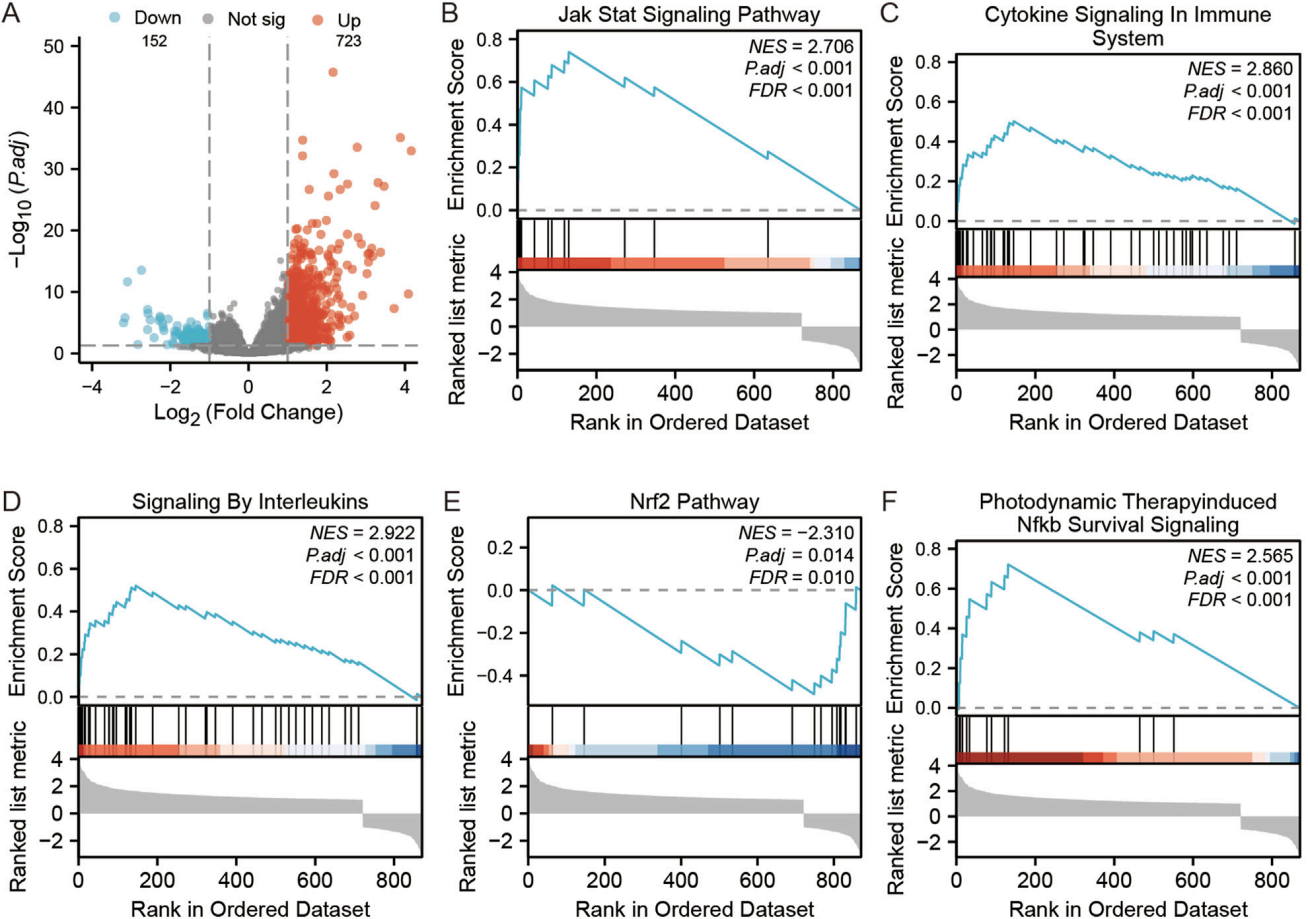
GSEA analysis of STC1-related differentially expressed genes. **(A)** Volcano map of STC1-related differentially expressed genes. **(B–F)** GSEA pathway enrichment results of STC1-related differentially expressed genes.

There were altogether 875 differentially expressed genes detected for screening, consisting of 723 with upregulation while 152 with downregulation, comparing the STC1 high and low expression cohorts (Figure 9A). Additionally, Gene Set Enrichment Analysis (GSEA) was conducted, revealing significant associations of STC1 with several pathways, including “Signaling by Interleukins”, “Cytokine Signaling in Immune System”, “JAK-STAT Signaling Pathway”, “Photodynamic Therapy-Induced NF-κB Survival Signaling”, and “NRF2 Pathway” (Figures 9B–F; Supplementary Table 2).

## 4 Discussion

Several studies have reported that STC1 expression markedly increases within tumor tissues, which is linked to poor patient prognosis (Smyth et al., 2020; Hou et al., 2021; Shir et al., 2012; Su et al., 2015). Therefore, we conducted a comprehensive bioinformatics study for analyzing STC1 expression and its clinical value in GC. We concluded that high STC1 expression in tissues was related to dismal patient prognosis. Furthermore, patients with high STC1 expression in GC exhibited inferior clinicopathological characteristics compared to those with low expression (Fang et al., 2014). Multivariate and univariate Cox analyses also revealed STC1 as the prognostic biomarker for OS of GC cases. Previously, STC1 exerts an important effect on other cancer types. For instance, according to Costa et al., STC1 expression markedly increased within prostate cancer tissues, whereas cellular experiments showed a reduction in prostate cancer cell proliferation and an increased cell death rate with STC1 antibody interference (Costa et al., 2020). Finally, Wang et al. indicated that STC1 downregulated BCL2 and contributed to increased proliferation, invasion, and chemoresistance in GC cells under hypoxic conditions (Wang et al., 2019).

Our study determined that STC1 expression is positively correlated with numerous immune cell types, including Th1 cells, macrophages, neutrophils, effector memory T cells, natural killer cells, myeloid dendritic cells, mast cells, plasmacytoid dendritic cells, eosinophils, dendritic cells, immature dendritic cells, follicular helper T cells, CD56 dim natural killer cells, CD8^+^ T cells, γδ T cells, activated dendritic cells, and cytotoxic cells. Th1 cells differentiate from natural CD4^+^ Th0 cells dependent on cytokine, and their cytokine production can suppress the tumor-promoting microenvironment (Li et al., 2021). Neutrophils accumulate in inflammation sites, promoting cancer mainly through increasing DNA damage, angiogenesis, and immunosuppression. They also recruit macrophages and Treg cells, facilitating tumor growth and progression (Xiong et al., 2021; Zhou et al., 2016). Although NK cells can kill tumor cells via various mechanisms, their function is limited by the immunosuppressive TME (Ko et al., 2019). γδ T cells enable tumor cell killing, inducing CD4^+^ and CD8^+^ T cell differentiation and proliferation. Moreover, they relieve immunosuppression from Treg cells and prompt CD8^+^ T cells for producing cytotoxic effects against GC (Saura-Esteller et al., 2022; Mao et al., 2014). Antigen-presenting cells (APC), such as macrophages and dendritic cells, capture sufficient antigen from dead tumor cells via phagocytosis, activate T cells while initiating anti-cancer immunity, thus exerting an important effect on immune checkpoint therapy (Salmon et al., 2016), while tumor STC1 impairs membrane CRT-mediated by reducing the levels of cell membrane calreticulin (CRT) and thus APC phagocytosis and T cell activation, inhibiting tumor immunity and immunotherapy (Lin et al., 2021). STC1 expression is also linked with the expression of T-cell depletion markers, like PD-1, PD-L1, CTLA-4, LAG3, TIM3, and GZMB, implying its role in immune regulation in GC. Sustained antigen exposure during chronic infection and cancer causes T-cell exhaustion, leading to poor response to immunotherapy. Blockading PD1 and CTLA4 could reinvigorate the immune response (Wherry, 2011). Our study found that STC1 expression correlates with T-cell exhaustion marker expression, such as PD-1, PD-L1, CTLA-4, LAG3, TIM3, and GZMB, indicating its role in immune regulation in GC. T-cell exhaustion is the decreased memory T cell function caused by sustained antigen exposure during chronic infections and cancer, leading to a poor response to immunotherapy. Blocking PD-1 and CTLA-4 can rejuvenate the immune response (Wang et al., 2017). Additionally, Treg cells, tumor-associated macrophages (TAMs), immature dendritic cells, plasmacytoid dendritic cells, and myeloid-derived suppressor cells (MDSCs) infiltration in TME promote T-cell exhaustion (Wang et al., 2017; Kersten et al., 2022; Kim et al., 2011). Our results suggest that STC1 expression has a significant association with infiltration degrees of multiple immune cells, indicating its involvement in TME regulation and T-cell exhaustion. Our scRNA-seq analysis further corroborates this perspective. The results demonstrate a co-expression pattern of STC1 with multiple T-cell exhaustion markers, particularly in T cells and epithelial cells. This co-expression pattern suggests that STC1 may play a significant role in the immunosuppressive TME. In studies of laryngeal squamous cell carcinoma (LSCC), STC1 has been shown to regulate the M2 polarization of TAMs and modulate immune cell function through metabolic reprogramming (Chen et al., 2025). STC1 is highly enriched in small extracellular vesicles (sEVs), and its knockdown can inhibit the metabolic reprogramming of TAMs into M2-like macrophages, thereby restoring CD8^+^ T cell function (Chen et al., 2025). These findings indicate that STC1 might be a key factor influencing T-cell exhaustion by modulating metabolic reprogramming and PD-L1 expression. The high expression of STC1 in GC is closely associated with T-cell exhaustion and poor prognosis.

Moreover, based on pathway enrichment, differentially expressed genes related to STC1 were significantly associated with signaling pathways such as JAK-STAT. Notably, JAK-STAT pathway is a complex pathway with dual functions of both anticancer and pro-cancer effects, which contributes to regulating activities of various immune cells and thus affects TME, ultimately influencing tumor development. The enrichment of the “Cytokine-cytokine receptor interaction” pathway also suggests that STC1 is involved in the communication network of cytokines. During tumor development, cytokines act as a bridge for the exchange of information between tumor cells and TIILs in TME, playing an important role in shaping TME (Sheu et al., 2008). Studies indicate that the activation of T cells can be attained by IFN-α and IFN-β as they promote the initiation of dendritic cells (DC), resulting in immediate and long-lasting immunity against cancer cellsStudies indicate that the activation of T cells can be attained by IFN-α and IFN-β as they promote the initiation of dendritic cells (DC), resulting in immediate and long-lasting immunity against cancer cells (Fuertes et al., 2013). IFN-γ can enhance the cytotoxicity and lysis of natural killer (NK) cells and activated CD8^+^ T cells (Takeda et al., 2017). Through the JAK-STAT pathway, IFN-α, IFN-β, and IFN-γ elicit an immune response against tumors by regulating downstream genes (Hassel et al., 2008; Michalska et al., 2018). Extensive cross-regulation exists between components of NF-κB and JAK/STAT pathways. NF-κB triggers expression of various inflammatory factors, which serves as an important transcription factor for different immune responses. It can induce malignant tumors and anti-tumor immunity through inflammation (Zhang et al., 2017).

In conclusion, STC1 exhibits high expression levels in GC tissues and independently predicts the prognosis of GC patients. Its expression correlates with TME cell infiltration and T-cell exhaustion. Nevertheless, the heterogeneity among tissue cells and GC patients, alongside the complexity of TME components, have hindered the clear elucidation of factors that impact GC progression. Hence, it is essential to investigate further the role of STC1 in the regulation of TME.

## 5 Limitations

This study has several limitations that should be considered. The data used for analysis were integrated from multiple publicly available databases, including TCGA, GTEx, and GEO. While these large-scale datasets are of significant value, the integration of data from different sources may introduce batch effects or biases due to variations in preprocessing methods and differences in patient demographics. These disparities could affect the consistency and generalizability of the results. Additionally, this study did not stratify GC samples based on molecular subtypes, such as Epstein-Barr virus (EBV)-positive or microsatellite instability (MSI)-high GC, which are known to exhibit distinct immune microenvironments. The immune characteristics of these subtypes may influence the role of STC1 in GC. Stratifying the samples by these subtypes would provide deeper insights into potential differences in STC1 expression and its association with immune infiltration across different GC subtypes. While this study examined the correlation between STC1 expression and tumor-infiltrating immune cells within the TME, it did not explore the spatial relationships or functional interactions between STC1 and immune cells. The spatial localization of immune cells relative to STC1 expression, as well as the potential functional effects of STC1 on immune cell activities, such as phagocytosis or antigen presentation, were not investigated. Future studies addressing these spatial and functional aspects will help elucidate the precise mechanisms by which STC1 modulates immune responses in GC.

## Data Availability

The datasets presented in this study can be found in online repositories. The names of the repository/repositories and accession number(s) can be found in the article/supplementary material.
